# Supplementary material for: Novel genes involved in the genetic architecture of temperament in Brahman cattle
Source: PLoS One. 2020 Aug 21;15(8):e0237825. doi: 10.1371/journal.pone.0237825 (PMC7446865; doi:10.1371/journal.pone.0237825)
Supplement: Attachment — Submitted filename: Response to reviewers.docx [file pone.0237825.s004.docx]

June 12, 2020

**Dear Editor:**

Here, you will find our responses to the reviewers’ questions. In general, to complete all of the requested revisions, necessary, major modifications in the methods, results and discussion sections of the manuscript were accomplished. In the former, we reanalyzed the physical position of significant SNPs based on bovine *Bos taurus* genome (ARS-UCD 1.2) and in the discussion we added information about how previously described genes in the human allowed us to infer their role in bovine temperament. All minor requirements were completed according to reviewer instructions.

Reviewer #1: Majors

1. As you wrote in L301-304, the aim of this manuscript was to identify the genes associated with temperament, such as stress response, innate fear, reaction to handling and aggressiveness. Considerations seemed to be insufficient in Discussion. You indicated the associations of candidate genes with results in other reports, but these associations were indirect and ambiguous. For example, in L241-248, the readers could not understand the relationship between the SNP and temperament (stress response, innate fear, reaction to handling and aggressiveness) even if “rare mutations and copy-number variations of human neurexin genes have been linked to autism and schizophrenia”. The authors should indicate the mechanism for mutation of neurexin genes induced autism and schizophrenia, and then the relationship between autism/schizophrenia and temperament (stress response, innate fear, reaction to handling and aggressiveness). As for other mutations, you should describe them.

**Authors:**  In L244-L255 of the revised manuscript, we included a more detailed description at the beginning of this discussion in order to introduce to the readers our inferences about how previously described genes in human disorders could be the basis to study their role in bovine temperament. It is worthy to notice that until now all association studies in humans and non-humans are allowing the scientist to explore and hypothesize about the genetic architecture of complex traits. The results of these GWAS investigations are the starting point to study at the molecular level how the discovered candidate genes are involved in expression of the trait. For most of these genes the mutation mechanisms are still unknown, but a wide number of studies support its association to expression of the disorder.

Minor: There were some typographical errors.

L87-8: add the approval number.

L185: insert a space between “0.266),” and “and”

L206-7: indicate not only web address but also references.

L213: add any references.

L245: add any references.

L247: add any references.

L286: add any references.

L288: add any references.

L253: polymorphismos -> polymorphisms

**Authors:**  We attended to all of the listed minor requirements according to reviewer‘s instructions.

Reviewer #2: A genome-wide association study identifies novel candidate genes related to cattle

1. L127-133: how was the genome-wide association study performed? Pedigree size? Model? Software?

**Authors:**  In the L122-L125 of the revised manuscript we include the requested information

2. L134-143: not clear why you chose these 14 SNP (I can guess why, but it can be not clear for all the readers); Furtherly, it is not clear how you estimated the LSM.

**Authors:**  After a Genome Wide Association Study analysis using a case control approach, these 14 SNPs were significantly associated indicating the putative position of QTLs for our assessed temperament traits (please see L130-131).

Least Square Means were estimated from the mixed model fitted for each marker included in the model, a complete description was included (please see L144-146).

3. L135 and L145: at this point I do not really understand if you consider all the 3 traits (as in L135) or only EV (as in L145). It seems that you provide results only for EV, but you mention other 2 traits twice in the manuscript (e.g. L95-103).

**Authors:**  We achieved the GWAS analysis with PLINK 1.9 software as cases and controls (please see L122-125); we classified the population into calm and temperamental groups from exit velocity (EV; please see L106-L118), although PLINK already shows that there is a significant association between the significant SNPs and bovine temperament, we also included an additional association analysis (as is described on L138-146) to estimate the quantitative effect of these markers on exit velocity, and other temperament parameters that were available for this population (i.e., Pen Score and Temperament Score).

4. Apart from this, I also strongly suggest to use the last available and more accurate assembly for *Bos taurus*, the ARS-UCD 1.2, for the codification.

**Authors:**  As is described in L131-L134, we reanalyzed the physical position of significant SNPs based on the bovine *Bos taurus* genome (ARS-UCD 1.2), because of this update additional precision in the number of the described genes were needed in the results section and in some tables. All changes were highlighted.

Few minor comments:

L61: ‘indirect estimation of heritability’

L64: define the abbreviation ‘SNP’ here

L77: the reader may get confused at this point; I recommend to write ‘in two extreme groups, i.e. ‘calm’ and ‘temperamental’ animals.’

L80: ‘show better growth performance than’

L82: ‘newborn animals’ rather than ‘neonates’

L82-83: ‘would help Brahman farmers in decision making and will improve human safety and animal welfare’

L95: delete reference name

L95-103: a bullet point would be easier to follow; anyway, the list and description provided may fit better in the introduction rather than in M&M, since you use only EV in this study

L108: be consistent with the use of ‘EV’ throughout the text; sometimes I see the full name

L125: rephrase as ‘individuals with more than 10% missing genotypes were also excluded’

L126: provide the % of females and the % of males defined as ‘calm’

**Authors:**  We attended to all of the listed minor requirements according to reviewer instructions

Many thanks in advance for your kind attention.

Sincerely yours,

**Ana María Sifuentes-Rincón**

Corresponding author

asifuentes@ipn.mx
