# Supplementary material for: Novel genes involved in the genetic architecture of temperament in Brahman cattle
Source: PLoS One. 2020 Aug 21;15(8):e0237825. doi: 10.1371/journal.pone.0237825 (PMC7446865; doi:10.1371/journal.pone.0237825)
Supplement: Attachment — Submitted filename: 2Response to reviewrs.docx [file pone.0237825.s005.docx]

Dear Editor:

Here, you will find our responses to Reviewer #2 changes and suggestions.

In general, minor changes in format were necessary and were attended as is detailed. The major review was a change in the manuscript title, however, we are agreeing with the Reviewer ´s suggestion and accepted the new title.

Reviewer #2: Overall, authors answered to my questions and my major concern was solved. I am satisfied with the new version.

Some minor changes and suggestions

• Table 3: Least square means would be easier to read if you add a row between different SNP in order to separate them. In addition, you can remove the SE, as you already have the letters of the t-test. Note that usually superscript letters are adopted for this purpose.

**Authors:**  *We attended the requirements according to reviewer recommendation.*

• For consistency, consider to use always ‘BTA’ instead of ‘Chr’ or ‘chromosome’ throughout the whole manuscript (see line 69, 71, 72, 74, 150, 204, 205, 206…)

**Authors:**  *We attended the requirements according to reviewer recommendation (see Table 1 and lines 148, 151, 162, 168, 169, 171, 172, 202-204, 206, 207, 470).*

• Line 133: ‘on the last available Bos Taurus genome’

**Authors:**  *We attended the requirements according to reviewer recommendation (see line 130).*

• Lines 142-143: I recommend to briefly explain why you used year of birth as random effect

**Authors:**  *A brief justification was included. Please see L139-142.*

• Table 1: position in Mb is sufficient

**Authors:**  *We attended the requirements according to reviewer recommendation.*

• Table 2: ‘gene ID’ is not needed

**Authors:**  *We attended the requirements according to reviewer recommendation.*

• Should sub-titles be smaller than the main section title? (see for example “Discussion” and Line 202 or Line 242)

**Authors:**  *We attended the requirements according to reviewer recommendation and change some subtitles. Please see lines 116, 179, 200, 240.*

• Consider to change the title as ‘Novel genes involved in the genetic architecture of temperament in Brahman cattle’ …I think it sounds more appealing

**Authors:**  *We appreciate the suggestion and agree with the proposed title, it was changed according to reviewer recommendation*

• It makes sense to add the (phenotypic and genetic) correlations available in the literature for cattle exit velocity, pen score, and temperament score

**Authors:**  *Required information was included. Please see L105-106.*
